# Vagus nerve stimulation for refractory posttraumatic epilepsy: Efficacy and predictors of seizure outcome

**DOI:** 10.3389/fneur.2022.954509

**Published:** 2022-07-28

**Authors:** Mengyi Guo, Jing Wang, Chongyang Tang, Jiahui Deng, Jing Zhang, Zhonghua Xiong, Siqi Liu, Yuguang Guan, Jian Zhou, Feng Zhai, Guoming Luan, Tianfu Li

**Affiliations:** ^1^Beijing Key Laboratory of Epilepsy Research, Department of Brian Institute, Center of Epilepsy, Beijing Institute for Brain Disorders, Sanbo Brain Hospital, Capital Medical University, Beijing, China; ^2^Department of Neurology, Center of Epilepsy, Beijing Institute for Brain Disorders, Sanbo Brain Hospital, Capital Medical University, Beijing, China; ^3^Beijing Key Laboratory of Epilepsy Research, Department of Neurosurgery, Center of Epilepsy, Beijing Institute for Brain Disorders, Sanbo Brain Hospital, Capital Medical University, Beijing, China

**Keywords:** posttraumatic epilepsy, vagus nerve stimulation, efficacy, predictor, interictal epileptic discharges

## Abstract

**Background:**

Traumatic brain injury (TBI) has been recognized as an important and common cause of epilepsy since antiquity. Posttraumatic epilepsy (PTE) is usually associated with drug resistance and poor surgical outcomes, thereby increasing the burden of the illness on patients and their families. Vagus nerve stimulation (VNS) is an adjunctive treatment for medically refractory epilepsy. This study aimed to determine the efficacy of VNS for refractory PTE and to initially evaluate the potential predictors of efficacy.

**Methods:**

We retrospectively collected the outcomes of VNS with at least a 1-year follow-up in all patients with refractory PTE. Subgroups were classified as responders and non-responders according to the efficacy of VNS (≥50% or <50% reduction in seizure frequency). Preoperative data were analyzed to screen for potential predictors of VNS efficacy.

**Results:**

In total, forty-five patients with refractory PTE who underwent VNS therapy were enrolled. Responders were found in 64.4% of patients, and 15.6% of patients achieved seizure freedom at the last follow-up. In addition, the responder rate increased over time, with 37.8, 44.4, 60, and 67.6% at the 3-, 6-, 12-, and 24-month follow-ups, respectively. After multivariate analysis, generalized interictal epileptic discharges (IEDs) were found to be a negative predictor (OR: 4.861, 95% CI: 1.145–20.632) of VNS efficacy.

**Conclusion:**

The results indicated that VNS therapy was effective in refractory PTE patients and was well tolerated over a 1-year follow-up period. Patients with focal or multifocal IEDs were recognized to have better efficacy after VNS therapy.

## Introduction

Traumatic brain injury (TBI) is an important factor that leads to morbidity and mortality and results in reduced quality of life and lifespan of patients (1). Posttraumatic epilepsy (PTE), a common and debilitating consequence of TBI, accounts for 20% of symptomatic epilepsy cases in the general population and affects up to half of surviving soldiers with penetrating injuries (2, 3). The risk of PTE is highest within 2 years after injury and is closely associated with the severity of TBI (the relative risk for mild, moderate, and severe TBI is 1.5, 2.9, and 17.0, respectively) (4). PTE occurs mainly in young people, interfering with their most productive years and consequently requiring costly social care (5). Unfortunately, patients with PTE are usually resistant to antiepileptic drugs and have unsatisfactory surgical outcomes due to poor localization of the epileptic focus (6). Therefore, it is urgent to improve the therapeutic strategy for PTE.

Vagus nerve stimulation (VNS) is a safe and effective therapy for reducing seizures in patients with medically refractory epilepsy (7). Since it received U.S. Food and Drug Administration approval in 1997, VNS therapy has been administered to more than 100,000 patients (8). As reported, approximately 50% of patients with epilepsy benefit from a ≥50% reduction in seizure frequency after 1 year of VNS implantation, with an increasing benefit over time (9). In addition, VNS is effective for some specific conditions of epilepsy, such as genetic generalized epilepsy (10), Lennox–Gastaut syndrome (11), and refractory postencephalitic epilepsy (12). The efficacy of VNS on PTE has also been suggested (13, 14). In patients with PTE due to severe brain injury, VNS is a helpful treatment modality to reduce seizure frequency (13). Compared with patients with non-PTE, those with PTE have a greater reduction in seizure frequency (73% vs. 57%) at 24 months after VNS therapy (14). Thus, for patients with medically refractory PTE who are not good candidates for resection, VNS could be considered a promising therapeutic strategy in the clinic.

Predictors of VNS efficacy have been researched for several years (9). In addition to epilepsy duration, interictal epileptic discharges (IEDs), the implant age of patients, and some novel potential predictors, including brain connectomic profiling (8), heart rate variability (15), and genetic variations of adenosine kinase (16), have been suggested for the prediction of VNS efficacy in recent years. At present, there are no relevant studies on potential predictors of VNS efficacy in PTE. This study aimed to demonstrate VNS efficacy in 45 patients with refractory PTE and to initially evaluate the potential predictors of VNS efficacy.

## Methods and materials

### Definition of refractory posttraumatic epilepsy

Posttraumatic epilepsy referred to recurrent, unprovoked, and spontaneous seizures following TBI (17). The first posttraumatic seizures were classified as follows (17): (1) immediate seizures, which occur within 24 h after brain injury; (2) early seizures, which occur within 1 week after brain injury; and (3) late seizures, which occur more than 1 week after brain injury and constitute the diagnosis of PTE. TBI in the research was diagnosed according to a definite history and magnetic resonance imaging (MRI) results showing clear evidence of traumatic brain injury. Patients who reported other definite epileptic etiologic factors (brain tumors, traumatic brain injuries, chromosome disease, etc.) were excluded.

Refractory PTE was defined as “failure of adequate trials of two tolerated, appropriately chosen and used antiepileptic drug schedules (whether as monotherapies or in combination) to achieve sustained seizure freedom” for PTE patients.

### Patient selection

We retrospectively studied VNS efficacy in patients with refractory PTE from Sanbo Brain Hospital, Capital Medical University, between September 2008 and April 2021. In our comprehensive epilepsy center, each patient was preoperatively evaluated through magnetic resonance imaging (MRI), video electroencephalography (VEEG), and, in some patients, positron emission tomography-computed tomography (PET-CT), neuropsychological assessment, and magnetoencephalography (MEG). All patients were evaluated at a multidisciplinary team (MDT) conference to determine treatment strategies. The decision of PTE patients who were suitable for VNS therapy was based on our previous strategy (18). VNS was recommended for PTE patients in the following conditions: (I) patients whose epileptogenic focus could not be precisely localized; (II) patients with epileptogenic focus involved in the eloquent areas; (III) patients who did not accept surgical resection; and (IV) patients with early surgical failure. All patients recruited in this study were followed up for at least 1 year. SEEG and the Wada test would be conducted to determine whether the epileptic foci were involved in eloquent areas.

### Ethical standards

This study conformed with the World Medical Association Declaration of Helsinki published on the website of the *Journal of American Medical Association* and was approved by the Ethics Committee of Sanbo Brain Hospital, Capital Medical University (SBNK-2017-15-01). Written informed consent was obtained from all patients or their guardians.

### Clinical data collection

The medical history of patients was collected, including sex, age of epilepsy onset, age of VNS implantation, epilepsy duration, predominant type and frequency of seizures, number of preoperative AEDs, preoperative neurological deficit, history of status epilepticus (SE), spatial distribution of EEG, and brain MRI. Details of TBI were also obtained, including age at TBI, type of TBI, treatment of TBI, and interval between TBI and the first seizure.

Seizure type in this study was defined as the most frequent seizure type of each patient based on the medical documents recorded by the physician. The predominant seizure types were classified as “focal onset” and “generalized onset” based on the 2017 ILAE Classification of Epilepsy (19). Modified from our previous study (12), the monthly frequency of seizures was categorized into “ <30 times” and “≥30 times.”

### Preoperative evaluation

The standard 10–20 system of electrode placement was used for the 64-channel long-term video EEG monitoring in all patients for at least 24 h. The interictal epileptic discharges (IEDs) were classified as focal^*^ (including focal or multifocal epileptiform discharges only) and generalized according to our previous study (12): (1) focal^*^: including focal (in which the IEDs only involved one lobe or contiguous lobes) and multifocal (in which the IEDs involved ≥3 multiple lobes); (2) generalized: in which the IEDs were bilateral synchronous and generalized in both hemispheres. Similarly, for patients whose seizures were recorded, the ictal onset rhythms were also classified as focal^*^ (focal or multifocal) or generalized. Brain 1.5-T MRI scans with T1, T2, and fluid-attenuated inversion recovery (FLAIR) sequences were conducted in all included patients. Additional preoperative examinations included PET-CT, MEG, and SEEG. However, the results of those measurements were not evaluated in this study. All results of the preoperative evaluation were analyzed in detail by experienced neurologists, neurosurgeons, neuroradiologists, and electrophysiologists. VNS implantations were conducted by two neurosurgeons according to standard procedures (20), and the strategy for the adjustment of stimulation parameters was based on available guidelines (21).

### Programming strategy of VNS

The parameter setting of VNS was performed based on our previous programming strategy (18). The stimulation was initiated 7 days after implantation of the stimulator. During the initial parameter settings, 0.5 mA was set for the output current, 30 s was set for the signal on time, and 5 min was set for the signal off time. The signal frequency (30 Hz) and the pulse width (250 microseconds) were kept consistent, and the magnet current was set as 0.25 mA higher than the output current. During 1 month of discharge, the current intensity of efficacy was increased to 1.25–1.5 mA at the outpatient clinic. From then on, the parameters were modified to 0.25 mA every 3–6 months according to the improvement in seizure control and patient tolerance.

### Seizure outcome and follow-up

All included patients received follow-up for at least 1 year after VNS surgery. The outcomes of VNS therapy were determined by a questionnaire survey performed when patients were readmitted to the hospital for adjustment of the stimulus parameters or remote follow-up *via* online methods. The efficacy of VNS was evaluated according to our previous study (12). Responders were defined as patients with a reduction of over 50% in baseline seizure frequency of the predominant seizure type. Seizure freedom in this study was defined as complete freedom from all types of seizures at the last follow-up. VNS outcomes were collected at 3, 6, 12, and 24 months and at the last follow-up after VNS treatment. The last follow-up results were used to define the overall efficacy and potential predictors of VNS.

### Statistical analysis

All analyses were performed using SPSS Software version 23.0, and a *P*-value of < 0.05 was considered statistically significant. The mean ± SD was used to describe continuous variables with normal distribution, and the median (interquartile range, IQR) was used to describe continuous variables without normal distribution. Categorical variables were represented as frequencies. In univariate analysis, the Mann–Whitney U test was used for continuous variables without normal distribution, an independent *t*-test was used for continuous variables with normal distribution, and Pearson's chi-square or Fisher's exact test was used for categorical variables. Variables showing a *P*-value of < 0.05 in the univariate analysis were then entered into a logistic regression model.

## Results

### Demographic characteristics

**Figure 1:**
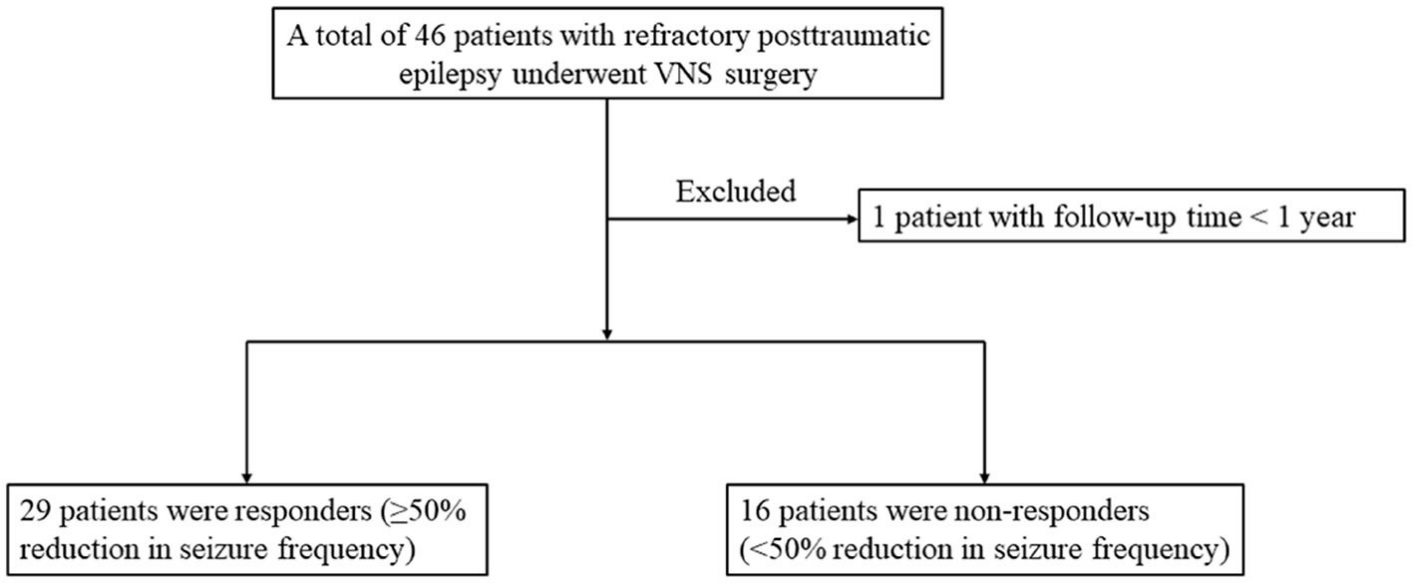
Flow chart for recruiting patients who satisfied the inclusion and exclusion criteria.

The overall flow of patient recruitment was shown in Figure 1. A total of 46 refractory PTE patients with VNS implantation were included, and 1 patient was excluded for follow-up time <1 year. This study was based on the remaining 45 patients with refractory PTE (35 men and 10 women) managed during 2008–2021. No serious adverse effects were reported in the enrolled patients.

Among the recruited refractory PTE patients, the median age of VNS implantation, age at seizure onset, and duration of seizures were 22.2 (IQR 14.3–33.5) years, 13.0 (IQR 8.0–25.0) years, and 4.0 (IQR 2.3–10.8) years, respectively. Four (8.9%) patients reported a history of SE, and seven (15.6%) patients had aura at the beginning of seizures. A preoperative neurological deficit was observed in 20 patients (44.4%): 14 (31.2%) had hemiparesis, 1 (2.2%) had aphasia, 3 (6.6%) had both hemiparesis and aphasia, 1 (2.2%) had ataxia, and 1 (2.2%) was reported as a persistent vegetative state. Other patient characteristics were shown in Table 1.

**Table 1:** Patients' demographic and clinical features and their relationship with VNS efficacy.

**Variable**	**Total (*n* = 45)**	**Responder (*n* = 29, 64.4%)**	**Non-responder (*n* = 16, 35.6%)**	***P*** **-value**
Male, *n* (%)	35 (77.8)	25 (86.2)	10 (62.5)	0.131
Age at VNS implantation, year old	22.2 (14.3, 33.5)	24.0 (13.6, 35.3)	20.0 (15.2, 31.0)	0.462
Age at seizure onset, year old	13.0 (8.0, 25.0)	14.0 (8.0, 26.8)	12.0 (4.3, 17.8)	0.235
Duration of seizures, year	4.0 (2.3, 10.8)	4.0 (2.0, 9.6)	4.7 (2.8, 16.8)	0.217
Age at TBI	11.0 (4.0, 24.0)	14.0 (4.5, 25.8)	7.5 (3.6, 15.5)	0.255
Interval between TBI and 1st seizure, year	0.8 (0.0, 3.5)	1.0 (0.0, 3.0)	0.6 (0.0, 4.0)	0.943
Type of the first posttraumatic seizure, *n* (%)				0.451
Immediate seizure	2 (4.4)	2 (6.9)	0 (0)	
Early seizure	11 (24.4)	6 (20.7)	5 (31.3)	
Late seizure	32 (71.2)	21 (72.4)	11 (68.7)	
Treatment of TBI				0.739
Craniotomy	24 (53.3)	16 (55.2)	8 (50.0)	
Conservative	21 (46.7)	13 (44.8)	8 (50.0)	
Type of TBI				0.172
MVA	15 (33.3)	10 (34.5)	5 (31.3)	
Blunt trauma	6 (13.3)	6 (20.7)	0 (0)	
Fall	19 (42.2)	11 (37.9)	8 (50.0)	
Unknown	5 (11.2)	2 (6.9)	3 (18.7)	
Monthly seizure frequency, n (%)				0.491
<30 times	33 (73.3)	20 (69.0)	13 (81.2)	
≥30 times	12 (26.7)	9 (31.0)	3 (18.8)	
Seizure type, *n* (%)				0.739
Focal onset	21 (46.7)	13 (44.8)	8 (50.0)	
Generalized onset	24 (53.3)	16 (55.2)	8 (50.0)	
Aura, *n* (%)				0.686
Yes	7 (15.6)	4 (13.8)	3 (18.8)	
No	38 (84.4)	25 (86.2)	13 (81.2)	
Types of AEDs				0.726
<3	35 (77.8)	23 (79.3)	12 (75.0)	
≥3	10 (22.2)	6 (20.7)	4 (25.0)	
Preop neurological deficit, *n* (%)	20 (44.4)	11 (37.9)	9 (56.3)	0.236
History of SE, *n* (%)	4 (8.9)	2 (6.9)	2 (12.5)	0.608
Spatial distribution of IEDs, *n* (%)				0.035*
Focal^†^	34 (75.6)	25 (86.2)	9 (56.3)	
Generalized	11 (24.4)	4 (13.8)	7 (43.7)	
Ictal onset rhythms of EEG, *n* (%)				0.144
Focal^†^	12 (26.7)	5 (17.2)	7 (43.8)	
Generalized	20 (44.4)	14 (48.3)	6 (37.5)	
Unknown	13 (28.9)	10 (34.5)	3 (18.8)	
Evidence of MRI pathology				0.826
Unilateral	15 (33.3)	10 (34.5)	5 (31.3)	
Bilateral	30 (66.7)	19 (65.5)	11 (68.8)	
Following time, year	3.0 (2.0, 4.5)	3.6 (2.0, 5.6)	2.7 (1.5, 3.0)	0.060

VNS, vagus nerve stimulation; TBI, traumatic brain injury; MVA, motor vehicle accident; AEDs, antiepileptic drugs; SE, status epilepticus; IEDs, interictal epileptiform discharges; EEG, electroencephalogram. Focal^†^, focal or multifocal. ^*^P <0.05.

### TBI and its treatment

The median age at TBI was 11.0 (IQR 4.0–24.0) years, and the interval between TBI and the first seizure was 0.8 (IQR 0.0–3.5) years. Nineteen (42.2%) patients were injured by falls, 15 (33.3%) patients by motor vehicle accidents, 6 (13.3%) patients by blunt trauma, and 5 (11.2%) patients by unknown factors. Out of the 45 patients with refractory PTE, 2 (4.4%) patients had immediate posttraumatic seizures, 11 (24.4%) patients had early posttraumatic seizures, and the remaining 32 (71.2%) patients had late posttraumatic seizures. Twenty-one (46.7%) patients recovered from TBI with conservative treatment alone. In the other 24 patients (53.3%), surgical treatments were performed: 8 (17.8%) patients underwent evacuation of an intracranial hematoma, 4 (8.9%) patients had decompressive craniectomy, 11 (24.4%) patients had both evacuation of an intracranial hematoma and decompressive craniectomy, and 1 (2.2%) patient had burr hole drainage. Of the 15 patients (33.3%) who underwent decompressive craniectomy, cranioplasty was performed in 4 (8.9%) patients before the preoperative assessment of epilepsy.

### Results of preoperative evaluation

**Figure 2:**
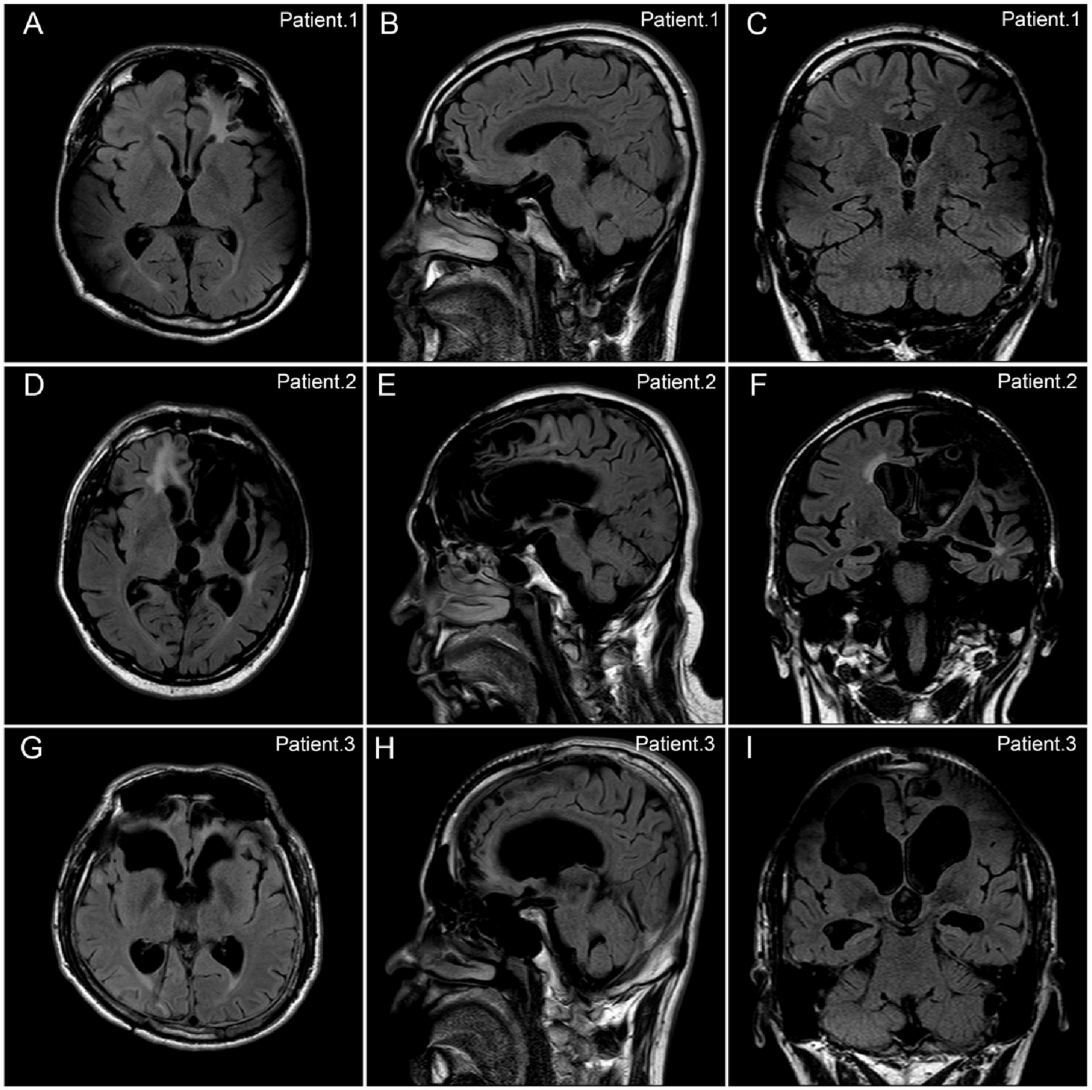
Representative FLAIR MR images of patients with refractory posttraumatic epilepsy. There were representative FLAIR MR images of three patients with refractory posttraumatic epilepsy (PTE) in the axial **(A,D,G)**, sagittal **(B,E,H)**, and coronal **(C,F,I)** planes. **(A–C)** Patient No.1. A 22-year-old boy with refractory PTE due to motor vehicle accident. The encephalomalacia was observed in bilateral frontal, parietal, and temporal lobes. The patient got seizure freedom after 2 years following the VNS therapy. **(D–F)** Patient No.2. A 51-year-old man with refractory PTE due to blunt trauma. The encephalomalacia was observed in the left frontal, temporal, and insula lobes, as well as in the right frontal lobe. The patient got a 75% reduction in seizure frequency after 7.5 years following the VNS therapy. **(G–I)** Patient No.3. A 26-year-old woman with refractory PTE due to motor vehicle accident. The encephalomalacia was observed in the bilateral frontal lobes. The patient got a 75% reduction in seizure frequency after 8 years following the VNS therapy.

**Figure 3:**
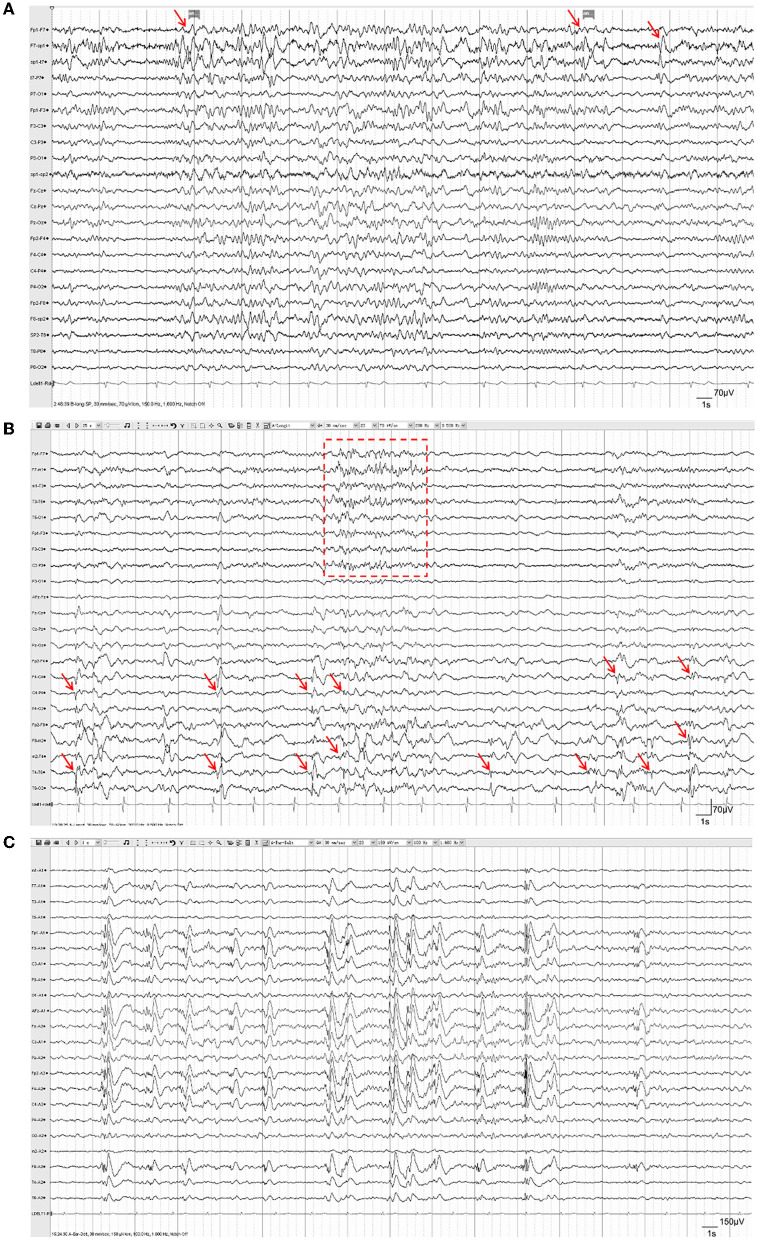
Representative EEG of patients with refractory posttraumatic epilepsy. **(A)** Focal IEDs. Spike-slow-wave discharges were observed in the left temporal lobe (F7, SP1, T7; red arrows), accompanied by increased irregular slow-wave discharges. **(B)** Multifocal IEDs. Intermittent spike-slow-wave discharges were observed mostly in the right central, parietal, and temporal lobes (F4, C4, P4, F8, M2, T4; red arrows), as well as in multifocal areas of the left hemisphere (red box); **(C)** Generalized IEDs. Generalized spike-slow-wave discharges were observed in both hemispheres synchronously and symmetrically.

Brain MRI results were reviewed in all patients. Encephalomalacia or focal cerebral atrophy was observed on preoperative MRI in all patients. Lesions in 15 (33.3%) patients involved only one hemisphere, and lesions in the other 30 (66.7%) patients involved both hemispheres. Representative MR images of three patients were shown in Figure 2. During scalp EEG monitoring, IEDs were observed in all patients: 34 (75.6%) were focal^*^(focal or multifocal) and 11 (24.4%) were generalized. The representative results of different types of IEDs are shown in Figure 3. Seizures were recorded in 32 (71.1%) patients, 12 (26.7%) of whom had focal^*^(focal or multifocal) epileptic discharges and 20 (44.4%) of whom had generalized epileptic discharges.

### Outcomes of VNS

The median time of the last follow-up was 3.0 (IQR 2.0–4.5) years for all participants, ranging from 1.0 to 11.7 years. At the last follow-up, seizures were reduced in 31 (68.9%) refractory PTE patients, and the median percent decrease in seizure frequency in 45 refractory PTE patients was 75.0 (IQR 0.0–100.0). There were 29 (64.4%) patients who had a seizure frequency reduction ≥50%, and seizure freedom occurred in 7 (15.6%) patients. McHugh and modified Engel seizure outcome classifications were used to evaluate the last follow-up outcomes (Table 2). The modified Engel scale found that of the 45 patients with refractory PTE, 7 (15.6%) patients were class I, 10 (22.2%) patients were class II, 12 (26.6%) patients were class III, and 16 (35.6%) patients were class IV. The McHugh scale showed that of the 45 patients, 22 (48.9%) patients were class I, 7 (15.6%) patients were class II, 2 (4.4%) patients were class III, and 14 (31.1%) patients were class IV–V.

**Table 2:** Seizure outcomes evaluated by modified Engel and McHugh classifications at the last follow-up (≥1 year).

**Class**	**Modified Engel Description**	**No. of Pts (%)**	**McHugh Description**	**No. of Pts (%)**
I	Seizure-free; rare, nondisabling SPS	7 (15.6)	80–100% reduction in seizure frequency	22 (48.9)
II	>90% reduction in seizure frequency; rare CPS	10 (22.2)	50–79% reduction in seizure frequency	7 (15.6)
III	50–90% reduction in seizure frequency	12 (26.6)	<50% reduction in seizure frequency	2 (4.4)
IV	<50% reduction in seizure frequency	16 (35.6)	Magnet benefit only	0
V	/	/	No improvement	14 (31.1)

*SPS, simple partial seizure; CPS, complex partial seizure; Pts, patients*.

**Figure 4:**
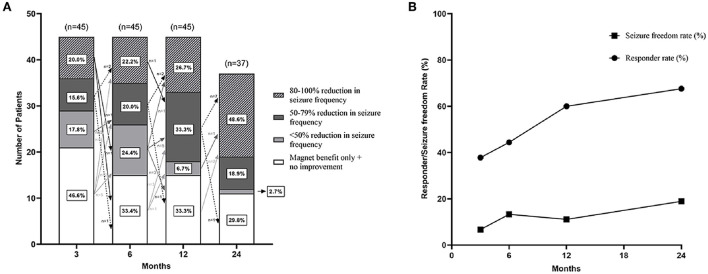
Seizure outcomes of patients with refractory posttraumatic epilepsy after VNS. **(A)** There were seizure outcomes at 3-, 6-, 12-, and 24-month follow-up after VNS therapy with McHugh outcome classification. Arrows indicated changes in VNS effectiveness between follow-ups. Figure **(B)** showed that the responder rate and seizure freedom rate gradually increased over time.

After VNS therapy, the outcomes of the 45 recruited refractory PTE patients were shown at the 3-, 6-, and 12-month follow-ups, and the outcomes of only 37 patients were shown at the 24-month follow-up (Figures 4A,B). The detailed assessments of VNS outcomes based on the McHugh description at different follow-up time points were shown in Figure 4A. Both the rates of responder and seizure freedom were found to gradually increase over time (Figure 4B). At 3, 6, 12, and 24 months of follow-up, the number of responder patients was 16 (35.6%), 19 (42.2%), 27 (60%), and 25 (67.6%), respectively; the number of patients with seizure freedom was 3 (6.7%), 5 (11.1%), 5 (11.1%), and 7 (18.9%), respectively; and the median percent of decrease in seizure frequency of 45 refractory PTE patients was 25.0 (IQR 0–50.0), 33.3 (IQR 0–70.8), 50.0 (IQR 0–87.4), and 75.0 (IQR 0–100.0), respectively.

The changes in VNS efficacy between follow-ups were shown in Figure 4A. Sixteen (35.6%) patients got response at 3 months after VNS therapy; among them, 3 (6.7%) patients adversely became non-responders at 6 months, 1 (2.2%) patient became a non-responder at 12 months, and 1 (2.2%) patient became a non-responder at 24 months. Out of the 29 (64.4%) non-responders at 3 months, 6 (13.3%) patients responded at 6 months, 9 (20.0%) patients responded at 12 months, 2 (4.4%) patients responded at 24 months, and 1 (2.2%) patient responded over 2 years (4 years) after VNS therapy. Besides, one non-responder at 6 months became a responder at 24 months, and one responder at 12 months became a non-responder at 1.5 years after VNS therapy. At the last follow-up, seven (15.6%) patients in the study population achieved seizure freedom; among them, five patients achieved seizure freedom within 1 year after VNS therapy, and two patients achieved seizure freedom at the 2-year follow-up.

### Analysis of prognostic factors for VNS efficacy

In the univariate analysis (Table 1), only the spatial distribution of IEDs was associated with seizure outcome. The factor was then entered into the logistic regression model. After analysis, generalized IEDs (OR: 4.861, 95%CI: 1.145–20.632) were found to be a negative predictor of VNS efficacy in refractory PTE. At the last follow-up (≥1 year), the responder rates of patients with focal^*^(focal or multifocal) IEDs and generalized IEDs were 73.5 and 36.4%, respectively.

Univariate analysis of other factors, such as sex, age at VNS implantation, age at seizure onset, duration of seizures, monthly seizure frequency, and MRI features, as well as the detailed factors of TBI, did not reveal significant differences between the two groups of responders and non-responders.

## Discussion

Traumatic brain injury is a common cause of epilepsy, and some patients with refractory PTE may be good candidates for surgical treatment (22). However, a proportion of PTE patients are unsuitable for the resective surgery. For those patients, VNS is an available neuromodulation strategy to provide therapeutic benefit (13, 14). In this study, we reviewed the clinical data of VNS efficacy for refractory PTE with a minimum follow-up of 12 months. Out of 45 patients with refractory PTE, 31 (68.9%) patients reported a reduction in seizure frequency, 29 (64.4%) patients achieved a reduction of over 50%, and 7 (15.6%) patients achieved seizure freedom after VNS surgery. As previously reported, approximately 45–65% of patients achieved a 50% or more reduction in seizure frequency (7), with patients rarely attaining complete seizure freedom (<5%) (9). Compared with other types of epilepsy, the results demonstrated a higher responder rate (68.9 vs. 45%) and a higher seizure freedom rate (15.6 vs. 5%) in refractory PTE patients after VNS therapy. This difference was in line with other observations suggesting that refractory PTE patients obtained even greater clinical benefit from VNS therapy than patients with refractory epilepsy unrelated to trauma (13, 14, 23). In addition, during the follow-up time ranging from 1 to 11.7 years among the 45 patients, no severe adverse effects were reported. Thus, the significant efficacy and safety of VNS therapy for refractory PTE were demonstrated. Furthermore, VNS efficacy has been reported to gradually increase over time in other refractory epilepsies (9, 24), and this study obtained similar results. At 3, 6, 12, and 24 months after device implantation for refractory PTE patients, the responder rates were 37.8, 44.4, 60, and 67.6%, respectively, and the seizure freedom rates were 6.7, 13.3, 11.1, and 18.9%, respectively. Thus, for patients with refractory PTE who are not good candidates for resection, VNS could be considered a promising therapeutic strategy.

In fact, reliable VNS efficacy on refractory PTE has been indicated previously (13, 14). Factors including the age at TBI, type of TBI, injury severity, hematoma, and craniectomy have been suggested to be associated with the development of PTE (25, 26). However, the predictors of VNS efficacy are still unknown. In this study, we first explored the potential predictors of VNS efficacy on refractory PTE *via* the surgical outcomes of 45 patients who received VNS implantation. After multivariate analysis, generalized IEDs were found to be the only negative factor for VNS efficacy in refractory PTE patients (OR: 4.861, 95% CI: 1.145–20.632). Compared with generalized IEDs, patients with focal or multifocal IEDs before VNS surgery had a higher responder rate at the last follow-up (73.5 vs. 36.4%). Similar results have been reported in numerous studies of VNS therapy in other types of refractory epilepsy (27–30). In refractory postencephalitic epilepsy, patients with focal or multifocal IEDs were found to be more likely to respond after VNS implantation than those with generalized IEDs (OR = 14.961, *P* = 0.003) (12). During a study including 47 patients who had undergone VNS implantation at one center and had follow-up after at least 1 year, the absence of bilateral IEDs was found to be a positive predictor of a seizure-free outcome (27). A study that recruited 436 patients with treatment-resistant epilepsy also reported that focal EEG findings predicted improved seizure control when combined with VNS therapy (30). Therefore, the crucial role of EEG findings in the prediction of VNS efficacy for epilepsy has been confirmed. In patients with refractory PTE, those with focal or multifocal IEDs were more likely to achieve favorable outcomes after VNS therapy.

Interestingly, similar to VNS efficacy, generalized IEDs were also a negative prognostic factor for resection surgery in epilepsy. As reported, bitemporal IEDs indicated bitemporal epileptogenicity in mesial temporal lobe sclerosis and were associated with a worse outcome than unilateral-temporal spike foci (31, 32). The most important reason may be that generalized IEDs represent an extended epileptogenic region or greater epileptogenicity as generalized IEDs are usually associated with a generalized seizure onset zone, generalized seizure propagation, and greater seizure frequency (33, 34). Another explanation is that generalized IEDs arise from an interaction of multiple active foci (35). Thus, whether for VNS efficacy or resection surgery, EEG features are a reliable tool for assessing outcome prognosis. Our findings on the correlation between generalized IEDs and worse VNS outcomes support the assumption that generalized IEDs represent a higher degree of epileptogenicity.

The potential predictors of VNS on refractory epilepsy have been researched for several years (7, 23). In addition to EEG features, seizure duration, age of seizure onset, age of VNS implantation, and seizure type have all been suggested to be related to seizure outcomes after VNS therapy (7). In our previous study focusing on predictors of seizure outcome after resection surgery for PTE, seizure duration of <8 years was found to indicate a favorable surgical prognosis (22). However, those factors were not significantly different between responders and non-responders in refractory PTE patients in this study. This may be because of the relatively small cohort in our research; some clinical phenomena might have no chance of reaching statistical significance. In the future, more studies with larger sample sizes could further explore those factors. In addition, factors suggested to be associated with the development of PTE, such as the age at TBI, interval between TBI and the first seizure, type of TBI, injury severity, hematoma, and craniectomy (25, 26), may also be linked with VNS efficacy when the sample size is enlarged. In addition to these frequent clinical and electrophysiological features, some novel predictive factors have recently attracted attention. These factors include brain connectomic profiling (8), heart rate variability (15), and genetic variations (16), which are worthy of further exploration in refractory PTE patients who receive VNS therapy.

Due to varying degrees of brain damage, PTE patients often have other neurological and psychological sequelae, including depression, anxiety, neurocognitive deficits, and chronic headache (36, 37). Improvements in these sequelae, as well as the overall quality of life, are another important aspect of therapeutic counseling for refractory PTE. In this study, 44.4% of patients reported preoperative neurological deficits. However, the psychological sequelae and the VNS efficacy on those symptoms were not further analyzed. It is well documented that VNS therapy may have a direct positive effect on behavior, concentration, and affect, often independent of seizure reduction (38). The recognized therapeutic effects of VNS in drug-resistant depression, as well as anxiety disorders and chronic headache, have also been suggested (39–41). Thus, the potential benefits of VNS on psychological and neurological disorders in refractory PTE patients could be further explored in the future.

Seizure freedom is usually recognized as the main predictor of quality of life in patients with epilepsy. Unfortunately, complete seizure freedom was rarely attained (<5%) in epilepsy patients who received VNS therapy. Among 45 patients with refractory PTE in this study, 7 (15.6%) patients had seizure freedom at the last follow-up, which was higher than that reported in the general population of epilepsy. Despite a lack of neuropsychological assessments, this result further supported that refractory PTE patients may have better improvements in the overall quality of life than patients with other types of epilepsy.

Some limitations of this study should be recognized. First, the inherent biases of the retrospective study and the relatively small sample size could not be ruled out in this study, and more prospective studies with large samples are expected to make the conclusions clearer in the future. Second, the severity (mild/moderate/severe) of TBI evaluated by the Glasgow Coma Scale (GCS) was not included in the study due to the large part of missing data. The related information will be added in the future studies. Third, clinical assessments of emotion, neurological deficits, and overall life quality were not conducted in this study, all of which are important for the curative effect of patients with refractory PTE. Despite these limitations, this study indicates the efficacy of VNS in reducing seizure frequency in patients with refractory PTE. In addition, generalized IEDs may be independent predictors of VNS efficacy.

## Conclusion

Our data demonstrated that VNS therapy was effective in patients with refractory PTE and was well tolerated over a 1-year follow-up period. Patients with focal or multifocal IEDs were found to have better efficacy after VNS therapy.

## Data availability statement

The datasets presented in this article are not readily available because of ethical and privacy restrictions. Requests to access the datasets should be directed to TL, tianfuli@ccmu.edu.cn.

## Ethics statement

The studies involving human participants were reviewed and approved by this study conformed with World Medical Association Declaration of Helsinki published on the website of the Journal of American Medical Association and was approved by the Ethics Committee of Sanbo Brain Hospital, Capital Medical University (SBNK-2017-15-01). Written informed consent was obtained from the individual(s), and minor(s)' legal guardian/next of kin, for the publication of any potentially identifiable images or data included in this article.

## Author contributions

TL, GL, and MG contributed to the conceptualization. TL, GL, MG, JW, CT, JD, and JZha contributed to the methodology. MG, SL, and ZX contributed to the formal analysis and investigation. MG contributed to the writing (original draft preparation). TL, GL, MG, JW, CT, JD, JZha, MG, JZho, FZ, SL, and ZX contributed to the writing (review and editing). MG, JZho, and FZ contributed to the investigation. All authors contributed to manuscript revision, and read and approved the submitted version.

## Funding

This work was supported by the National Key Research and Development Program of China (No. 2021YFC2401203), the National Natural Science Foundation of China (Grant Nos. 81571275 and 11932003), and Sanbo Brain Hospital Capital Medical University (No. 2022ZZLX04).

## Conflict of interest

The authors declare that the research was conducted in the absence of any commercial or financial relationships that could be construed as a potential conflict of interest.

## Publisher's note

All claims expressed in this article are solely those of the authors and do not necessarily represent those of their affiliated organizations, or those of the publisher, the editors and the reviewers. Any product that may be evaluated in this article, or claim that may be made by its manufacturer, is not guaranteed or endorsed by the publisher.
